# Genome-wide analysis of tomato NF-Y factors and their role in fruit ripening

**DOI:** 10.1186/s12864-015-2334-2

**Published:** 2016-01-07

**Authors:** Shan Li, Ka Li, Zheng Ju, Dongyan Cao, Daqi Fu, Hongliang Zhu, Benzhong Zhu, Yunbo Luo

**Affiliations:** The College of Food Science and Nutritional Engineering, China Agricultural University, No. 17 Tsinghua East Road, Beijing, 100083 Peoples Republic of China

**Keywords:** Genome-wide analysis, NF-Y transcription factor, Ripening, VIGS, Tomato

## Abstract

**Background:**

Fruit ripening is a complex developmental process that depends on a coordinated regulation of numerous genes, including ripening-related transcription factors (TFs), fruit-related microRNAs, DNA methylation and chromatin remodeling. It is known that various TFs, such as MADS-domain, MYB, AP2/ERF and SBP/SPL family proteins play key roles in modulating ripening. However, little attention has been given to members of the large NF-Y TF family in this regard, although genes in this family are known to have important functions in regulating plant growth, development, and abiotic or biotic stress responses.

**Results:**

In this study, the evolutionary relationship between *Arabidopsis thaliana* and tomato (*Solanum lycopersicum*) *NF-Y* genes was examined to predict similarities in function. Furthermore, through gene expression analysis, 13 tomato *NF-Y* genes were identified as candidate regulators of fruit ripening. Functional studies involving suppression of *NF-Y* gene expression using virus induced gene silencing (VIGS) indicated that five *NF-Y* genes, including two members of the NF-YB subgroup (*Solyc06g069310, Solyc07g065500*) and three members of the NF-YA subgroup (*Solyc01g087240, Solyc08g062210, Solyc11g065700*), influence ripening. In addition, subcellular localization analyses using NF-Y proteins fused to a green fluorescent protein (GFP) reporter showed that the three NF-YA proteins accumulated in the nucleus, while the two NF-YB proteins were observed in both the nucleus and cytoplasm.

**Conclusions:**

In this study, we identified tomato *NF-Y* genes by analyzing the tomato genome sequence using bioinformatics approaches, and characterized their chromosomal distribution, gene structures, phylogenetic relationship and expression patterns. We also examined their biological functions in regulating tomato fruit via VIGS and subcellular localization analyses. The results indicated that five NF-Y transcription factors play roles in tomato fruit ripening. This information provides a platform for further investigation of their biological functions.

**Electronic supplementary material:**

The online version of this article (doi:10.1186/s12864-015-2334-2) contains supplementary material, which is available to authorized users.

## Background

Fleshy fruit ripening is a complex developmental process, that typically involves changes in texture, color and flavor [66]. The regulatory mechanisms underlying these changes, and that coordinate the up- or down-regulation of large numbers of genes, include many ripening-related transcription factors (TFs), fruit-related microRNAs that target some of these TFs, DNA methylation and chromatin remodeling [55]. Members of several families of TFs, such as the MADS-box, MYB, AP2/ERF and SBP/SPL families, participate in the transcriptional regulatory network that modulates ripening [27]*.* For example, studies of several ripening-related mutants of the fleshy fruit model species tomato (*Solanum lycopersicum*) [15, 20], including *ripening inhibitor (rin)* [75]*, non-ripening (nor)* [30] and colorless *non-ripening (cnr)* [21, 45], resulted in the identification of several ripening-associated TFs. The genes corresponding to these loci were found to encode fruit ripening related TFs, which have subsequently been identified as components of the transcriptional activation cascade that coordinates ripening [27]. Other TF families that participate in fruit ripening, included MYB, AP2/ETHYLENE RESPONSE FACTOR (ERF), HD-zip, basic helix-loop-helix (bHLH) and auxin response factors (ARFs) [27]. For example, SlMYB12 from tomato and MdMYB10 from apple have been shown to influence flavonoid and anthocyanin accumulation during fruit ripening [1, 3, 14], and a member of the AP2/ERF family, AP2a, which acts downstream of RIN, NOR, and CNR, is a negative regulator of tomato ripening [9, 28]. In addition, silencing of *LeHB1*, which encodes a tomato putative HD-zip protein, led to delayed ripening and reduced expression *LeACO1*, a gene that encodes an ACC oxidase, which is a key enzyme in the biosynthesis of the ripening associated hormone ethylene [40]. Moreover, members of both bHLH and ARF families have been shown to play diverse roles in fruit development and ripening [32, 72].

While various families of TFs are clearly associated with ripening regulation, there has been no such connection established for the large Nuclear Factor Y (NF-Y) TF family, despite its known functions in regulating plant growth, development, and abiotic or biotic stress responses [4, 36, 57]. NF-Y TFs, which are also known as heme activator proteins (HAPs) or CCAAT binding factors (CBFs), can be categorized as NF-YA (also known as HAP2 or CBF-B), NF-YB (HAP3 or CBF-A) and NF-YC (HAP5 or CBF-C) proteins [39], and they have been shown to bind to CCAAT boxes, which are thought to be present in approximately 30% of eukaryotic promoters [6, 47]. The NF-YB proteins lack a nuclear localization signal (NLS), and depend on an interaction with NF-YC proteins to ensure translocation to the nucleus. Upon arrival in the nucleus, a heterotrimer is formed, comprising the NF-YB and NF-YC heterodimer and NF-YA, which can bind to CCAAT boxes in the promoters or other regions of target genes, [17, 26].

In order to investigate the potential functions of NF-Y TFs in fruit ripening, we evaluated a total of 59 tomato *NF-Y* genes using a combination of bioinformatic analyses, and high-throughput functional screening, using virus-induced gene silencing (VIGS) [12]; an approach that has been widely used to study tomato fruit development and ripening [18, 53, 60]. We also describe a phylogenetic analysis of *NF-Y* genes from *Arabidopsis thaliana* and tomato, and an investigation of their chromosomal distribution, protein motif and exon/intron structure patterns. As a result of these analyses, we propose five candidate *NF-Y* genes that are likely involved in fruit ripening regulation.

## Results

### Identification, organization and structure of tomato *NF-Y* genes

A search of the Plant Transcription Factor Database (PlantTFDB, http://planttfdb.cbi.pku.edu.cn/) revealed a total of fifty-nine predicted tomato *NF-Y* genes that, based on the encoded subunits, included ten NF-YA, twenty-nine NF-YB and twenty NF-YC genes (Table 1). The physical map positions of the *NF-Y* genes on the tomato chromosomes were identified (Additional file 1: Figure S1), according to their ascending order of physical position (bp), from the short arm telomere to the long arm telomere. Of the fifty-nine *NF-Y* genes, fifty-seven could be mapped onto the twelve tomato chromosomes, with the exceptions being *Solyc00g107050* and *Solyc00g270510*. Chromosome 1 contained three *NF-YA* genes, while chromosomes 4 to 7 and chromosome 9 did not contain any genes from this sub-group. Chromosome 5 contained the largest number of *NF-YB* genes, seven, almost all of which were located in the upper part of the chromosome. A total of ten *NF-Y* genes were located on chromosome 5. Chromosome 3 had the largest number of *NF-YC* genes, six. It appears that the pattern of *NF-Y* genes across plant genomes is uneven and that the distribution varies among different species. For example, in common bean (*Phaseolus vulgaris*), *PvNF-Y* genes were mapped to 10 out of the 11 chromosomes [63] and the nine *PvNF-YA* genes were uniformly distributed, while five of the *PsNF-YB* genes were located on chromosome 7. Moreover, members of the *PvNF-YC* subfamily were found on five chromosomes, with one or two per chromosome [63].

**Table 1:** NF-Y transcription factors in tomato

Protein properties of tomato NF-Y TFs							
NF-YA Subunit				NF-YB Subunit			
Subunit	Gene ID	Length (AA)	pI	Subunit	Gene ID	Length (AA)	pI
NF-YA1	*Solyc01g008490*	312	7.01	NF-YB3	*Solyc04g054150*	188	8.18
	*Solyc11g065700*	300	7.23		*Solyc07g065500*	182	6.31
NF-YA3	*Solyc03g121940*	241	9.39		*Solyc12g006120*	202	6.36
	*Solyc12g009050*	253	8.86	NF-YB5	*Solyc01g067130*	138	5.96
NF-YA7	*Solyc02g069860*	208	7.84		*Solyc01g099320*	146	4.9
	*Solyc10g079150*	219	9.88		*Solyc06g009010*	178	5.4
NF-YA8	*Solyc08g062210*	325	9.07		*Solyc09g074760*	138	5.31
NF-YA9	*Solyc01g087240*	303	8.82	NF-YB6	*Solyc02g032180*	287	5.02
NF-YA10	*Solyc01g006930*	311	10.06		*Solyc02g032190*	95	4.84
	*Solyc10g081840*	154	10.93		*Solyc04g015060*	237	5.89
NF-YC Subunit					*Solyc05g005350*	219	4.18
Subunit	Gene ID	Length (AA)	pI		*Solyc05g005360*	224	4.27
NF-YC1	*Solyc03g110860*	230	5.04		*Solyc05g005380*	357	5.96
	*Solyc03g111450*	318	4.44		*Solyc05g005390*	238	5.01
	*Solyc03g111460*	288	8.2		*Solyc05g005440*	208	4.95
	*Solyc06g072040*	232	5.02		*Solyc05g015550*	219	5.34
NF-YC3	*Solyc08g007960*	124	6.94		*Solyc07g065570*	188	8.36
NF-YC4	*Solyc00g107050*	144	5.73		*Solyc07g065580*	178	9.61
	*Solyc02g021330*	153	4.67		*Solyc10g009440*	238	4.88
	*Solyc02g091030*	677	4.05		*Solyc11g012750*	152	6.23
	*Solyc03g110840*	162	5.3	NF-YB7	*Solyc12g027650*	208	7.96
	*Solyc03g110850*	163	5.1	NF-YB8	*Solyc04g009520*	86	5.88
	*Solyc03g111470*	273	8.08		*Solyc04g049910*	165	4.86
	*Solyc11g016910*	93	4.86	NF-YB10	*Solyc09g007290*	129	6.52
	*Solyc11g016920*	138	4.76	NF-YB11	*Solyc11g068480*	175	4.64
NF-YC9	*Solyc01g079870*	258	6.15	NF-YB13	*Solyc03g114400*	132	4.26
NF-YC10	*Solyc06g016750*	446	8.86		*Solyc06g069310*	131	4.25
NF-YC11	*Solyc01g096710*	276	5.17	Histone superfamily	*Solyc00g270510*	184	6.6
NF-YC13	*Solyc05g015330*	144	9.96		*Solyc05g005370*	224	4.33
HistoneH2A2	*Solyc05g014800*	135	10.94				
	*Solyc05g014830*	292	10.75				
Histone superfamily	*Solyc11g072150*	305	4.68				

*pI: Isoelectric point

We next analyzed the exon-intron structure of the tomato *NF-Y* genes (Additional file 2: Figure S2). The *NF-YA* genes had four to seven exons, and most (eight out of ten had five or six exons, while the majority of the *NF-YB* genes (twenty out of twenty-nine) had one or two exons. However, the structure of the *NF-YC* genes was more variable and thirteen out of twenty had one or two exons, while others had three to six exons, and one *NF-YC* gene, *Solyc02g091030*, had twenty-two exons. Overall, the gene structure analysis revealed that the members of the *NF-YA* family had a relatively consistent intron/exon organization (Additional file 2: Figure S2), while the *NF-YB* and *NF-YC* sub-groups were more variable among different members, which is similar to the organization reported for common bean *NF-Y* genes [63].

### Multiple alignment of NF-Y protein sequences and analysis of the evolutionary relationship between tomato and *A. thaliana* families

**Fig. 1:**
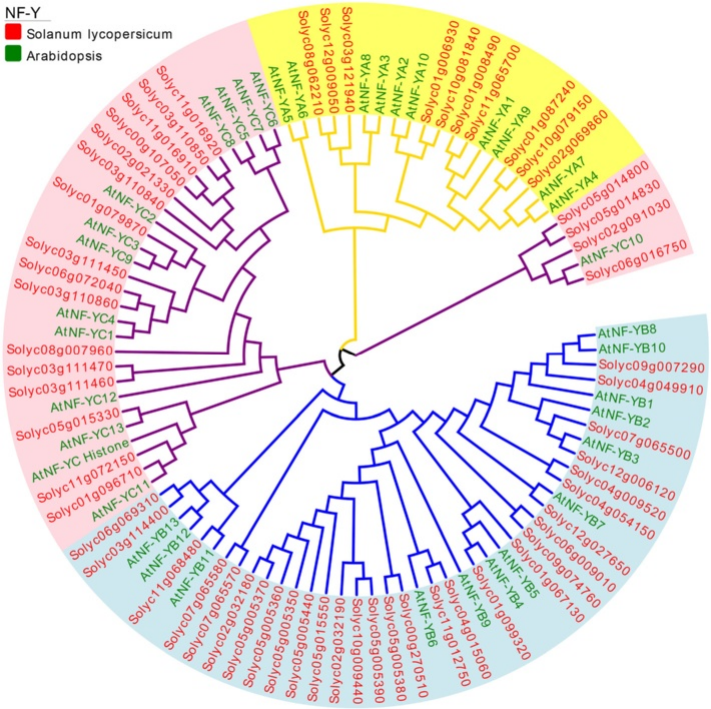
Phylogenetic analysis of tomato and *A. thaliana* NF-Y proteins. Phylogenetic analysis of 96 NF-Y proteins from tomato (59) and *A. thaliana* (37). Four branches were formed corresponding to genes with different subunits. Pink represents two branches of NF-YC, blue represents NF-YB and yellow represents NF-YA

Multiple sequence alignments were generated of proteins corresponding to members of the tomato and *A. thaliana* NF-Y subunit family (NF-YA, NF-YB, NF-YC). The *A. thaliana* NF-Y family was comprised of ten AtNF-YA, thirteen AtNF-YB and fourteen AtNF-YC proteins. Each sub-family was found to have one or more central core regions with extensive homologous motifs (Addtional file 3: Figure S3), that have been reported to be important in subunit interactions and DNA binding in yeast and mammals [29, 48, 70, 78]. The motifs of the tomato NF-Y proteins with putative functions in protein interactions and DNA binding were highlighted using the InterPro protein sequence analysis and classification database (Additional file 4: Fig. S4, http://www.ebi.ac.uk/interpro/). Finally, an un-rooted phylogenetic tree was created using the full length tomato and *A. thaliana* NF-Y protein sequences in order to gain insight into their evolutionary relationships and to assist with functional predictions (Fig. 1).

**Fig. 2:**
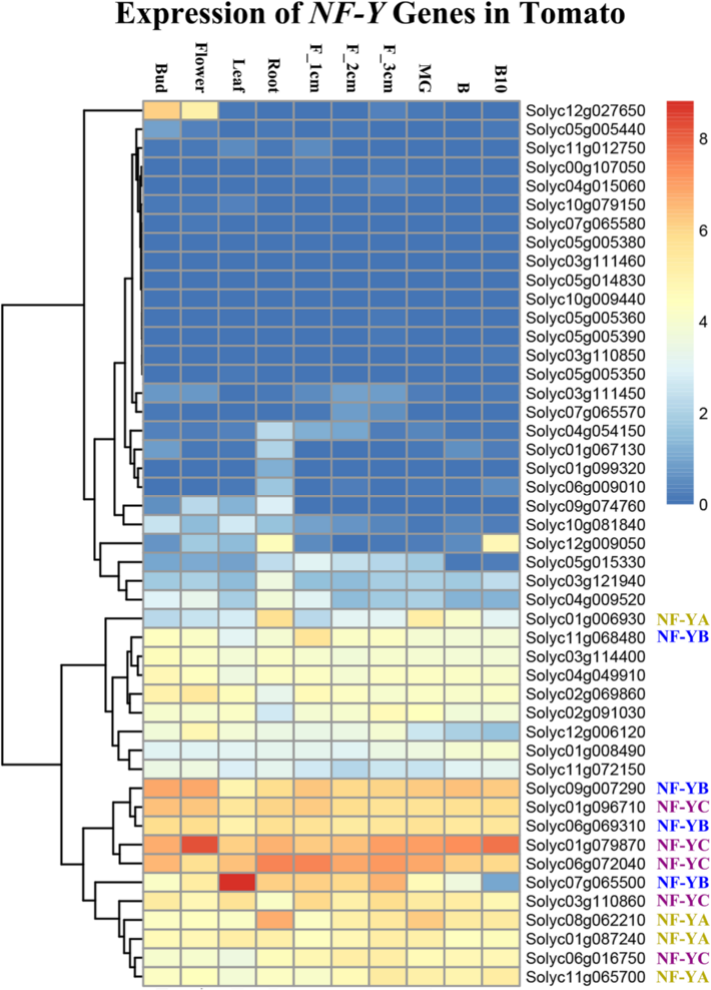
Expression patterns of *NF-Y* genes in various tomato organs. RNA-seq expression data corresponding to fifty-nine tomato *NF-Y* genes were retrieved from The Genome Consortium (TGC) datasets [65] for further analysis. Twelve genes with no detectable transcripts in any tissue/organ were excluded from the heat map. In the heat map, the RPKM (Reads Per Kilobase of exon model per Million mapped reads) values were transformed to log2(value + 1). Thirteen genes are shown labeled with their subunit on the right corresponding to genes targeted for subsequent analysis. The expression in various tomato organs is shown, including bud, flower, leaf, root, F_1cm (fruit with a 1 cm diameter), F_2cm, F_3cm, MG (Mature Green), B (Breaker), B10 (Breaker + 10 days)

The tomato and *A. thaliana* NF-YA proteins were variable in length, ranging from 154-325 and 186-340 amino acids (AAs), respectively (Table 1), and they were observed to share two characteristic conserved domains (Additional file 3: Figure S3). Previous studies with metazoans and yeast have indicated that the first twenty AA conserved domain is required for subunit interactions with NF-YB/YC, and that the other 21 AA domain is necessary for DNA binding and CCAAT sequence recognition specificity [46, 59, 78]. Outside the conserved regions, there was limited AA sequence conservation; however, the sequences were generally rich in Gln (Q) and Ser/Thr (S/T) residues, a feature that has been associated with promoting transcriptional activation [10, 11]. All the tomato NF-YA proteins contained predicted nuclear localization signals that were similar to those of the *A. thaliana* homologs (red boxes in Fig. 2; [26, 74]).

The NF-YB proteins from tomato and *A. thaliana* ranged from 86-357 and 139- 275 AA in length, respectively (Table 1). They were highly similar in a stretch of more than 90 AAs that is considered to be the central conserved domain involved in the interaction with the NF-YC and NF-YA subunits and DNA binding (Additional file 3: Figure S3). Outside this central region, the sequences were variable in both length and AA composition. Evolutionary analysis can be used to predict the function of members from the same clade based on the known function of one or more members of that clade. For example, AtNF-YB6 from *A. thaliana*, also known as LEC1-LIKE (L1L), is a regulator of embryo development, and *L1L* RNA accumulates in developing embryos [35]. The tomato *L1L* paralogs, *L1L1* to *L1L13*, have similar expression patterns in either seed or developing fruit, or both, and may therefore have a similar function [24].

The NF-YC sub-group contained a central domain of approximately 80 AAs that was highly conserved across the different members, and which has been shown to be important for both DNA binding and interactions between NF-Y subunits (Additional file 3: Figure S3) [64]. Most NF-YC proteins were enriched in Q residues (Additional file 3: Figure S3), a feature that has previously been noted in *A. thaliana* [69], *Brachypodium distachyon* [7] and other plant species [57]. In mammals and yeast, the Q rich regions of NF-YC proteins have been reported to function in transcriptional activation [10, 11]. The phylogenetic analysis identified two main clades, one containing AtNF-YC10 and four tomato NF-YC proteins, and another containing thirteen AtNF-YC and sixteen tomato NF-YC proteins (Fig. 1). Such gene structure analysis can often provide additional information regarding putative gene function [68] and here it was noted that three pairs of tomato *NF-Y* genes in the same clade had the same number of exons (Additional file 2: Figure S2).

### Expression patterns of *NF-Y* genes in different tomato organs and during fruit development

Tomato transcript expression (RNA-seq) data sets are publicly available, including expression in 10 tomato organ types: bud, flower, leaf, root, and fruit with 1 cm, 2 cm or 3 cm diameters, and at the Mature Green, Breaker, Breaker + 10 days stages of development and ripening [65]. These datasets were searched using the fifty-nine tomato *NF-Y* gene sequences and the results were used to construct a hierarchical clustering heat map (Fig. 2) displaying the expression patterns of forty-seven of the fifty-nine *NF-Y* genes. The other twelve *NF-Y* genes did not have any corresponding expression data and so were omitted from the analysis. Among remaining forty-seven observed *NF-Y* genes, more than half had low levels of transcripts, and these were enriched in top part of the heat map (Fig. 2). Specific *NF-Y* genes such as *Solyc12g027650* were more abundantly expressed in buds and flowers, but much lower transcript levels in fruit. In this study, we focused on *NF-Y* genes with relatively higher expressions in fruit during development and ripening, so thirteen members, comprising four *NF-YA*, four *NF-YB* and five *NF-YC* genes, were selected for further functional analysis (Table 2), which was also labeled with corresponding subunit in the heat map (Fig. 2).

**Table 2:** List of tomato NF-Y candidate regulators for fruit ripening

Gene ID	Subunits	Tissue-specific expression	Homologs in Arabidopsis	Functional annotation in Arabidopsis
*Solyc01g006930*	NF-YA	Root	AtNF-YA2,10	Seed germination [74]
*Solyc01g079870*	NF-YC	Various tissues	AtNF-YC3,YC9,YC2	Floral induction, ER stress, flowering [50, 56, 75, 76]
*Solyc01g087240*	NF-YA	Leaf	AtNF-YA4,YA7	Flower, ER stress [20]
*Solyc01g096710*	NF-YC	Bud	AtNF-YC11	
*Solyc03g110860*	NF-YC	Fruit	AtNF-YC1,YC4	Floral induction, Flowering, Germination [56, 75–77]
*Solyc06g016750*	NF-YC	Fruit	AtNF-YC10	
*Solyc06g069310*	NF-YB	Flower	AtNF-YB12,YB13	
*Solyc06g072040*	NF-YC	Fruit	AtNF-YC1,YC4	Floral induction, Flowering, Germination [56, 75–77]
*Solyc07g065500*	NF-YB	Leaf	AtNF-YB3,YB2	Flowering, root growth [18, 78, 79]
*Solyc08g062210*	NF-YA	Root	AtNF-YA3,YA8	Embryo development [80]
*Solyc09g007290*	NF-YB	bud	AtNF-YB8, YB10	
*Solyc11g065700*	NF-YA	Fruit	AtNF-YA1, YA9	Male gametogenesis, Embryogenesis, Seed development [81]
*Solyc11g068480*	NF-YB	Fruit	AtNF-YB11	

### Quantitative RT-PCR analysis of *NF-Y* gene expression in ripening tomato fruit and their responsiveness to ethylene and 1-MCP

**Fig. 3:**
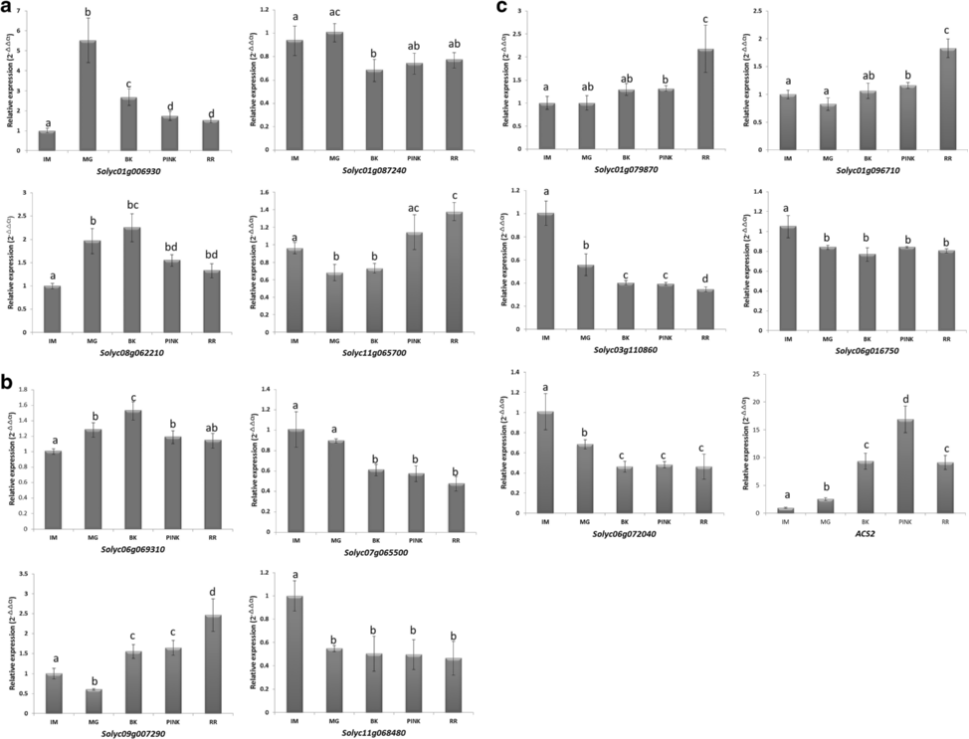
Expression of tomato *NF-Y* genes during fruit ripening. Figures are grouped into (**a**) NF-YA, (**b**) NF-YB, (**c**) NF-YC. Relative transcript levels are shown, as determined by quantitative real time RT-PCR, relative to the expression of the tomato *ACTIN* gene, expressed as 2^-△△Ct^ [43]. The stages of fruit ripening were: Immature (IM), Mature Green (MG), Breaker (BK), Pink (PK), and Red Ripe (RR). Significance values were based on comparisons of expression levels at different ripening stages with expression at the IM stage. Expression levels of the tomato *ACS2* gene were used as a positive control. Lowercase letters above the bars indicate values with a significant difference, which were determined by Student’s *t*-test (* = P < 0.05;** = P < 0.01)

Based on the RNA-seq expression profile analysis, thirteen tomato *NF-Y* genes were selected as candidates for fruit ripening regulation and their expression was further evaluated by real-time quantitative RT-PCR analysis at different fruit developmental stages: Immature (IM), Mature Green (MG), Breaker (BK), Pink (PK) and Red Ripe (RR). As shown in Fig. 3, the genes collectively displayed a range of expression patterns during ripening, and the changes in expression were in agreement with the RNA-seq profiles, other than extent of the changes for a couple of the genes. The expression patterns of some genes, such as *Solyc11g065700* (Fig. 3a), *Solyc09g007290* (Fig. 3b), *Solyc01g079870* and *Solyc01g096710* (Fig. 3c), were consistent with a role in promoting ripening since they showed up-regulated expression at onset of ripening. Others, such as *Solyc03g110860* (Fig. 3c) showed reduced expression during ripening, and *Solyc01g006930* (Fig. 3a) was down-regulated after the onset of ripening, although its expression increased at the BK stage, which might indicate a role in suppressing ripening. Two *NF-Y* genes showed a fluctuating expression patterns during development, including *Solyc06g069310* (Fig. 3b) and *Solyc01g087240* (Fig. 3a), although the pattern were opposite from each other. Other genes, including *Solyc08g062210* (Fig. 3a), *Solyc07g065500* and *Solyc11g068480* (Fig. 3b), *Solyc06g016750* and *Solyc06g072040* (Fig. 3c) showed minor changes once ripening had initiated.

**Fig. 4:**
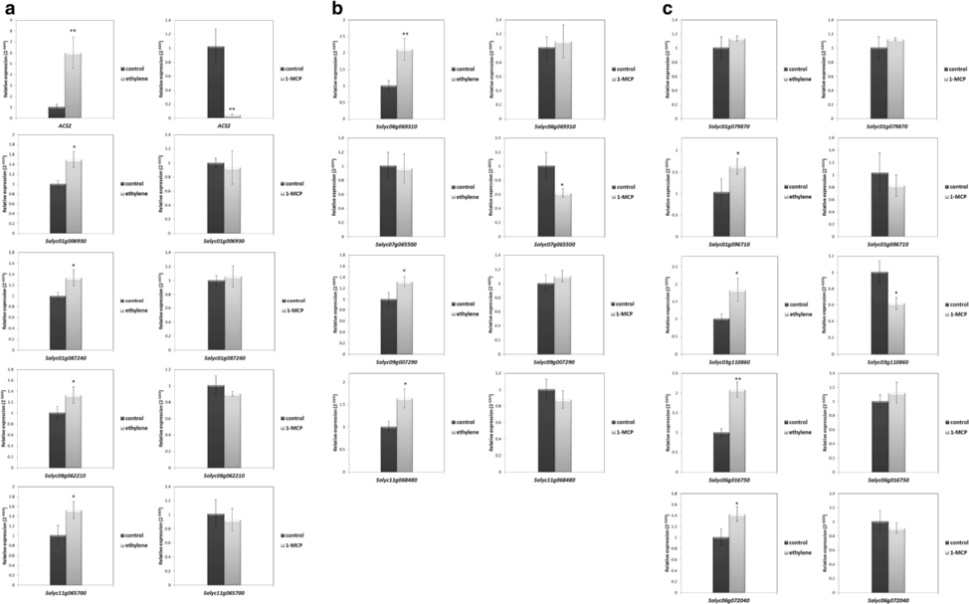
Expression of tomato *NF-Y* genes in fruit treated with ethylene or 1-MCP. Figures are grouped into (**a**) NF-YA, (**b**) NF-YB, (**c**) NF-YC. Fruit at the MG stage and after 24 h of treatment with ethylene or 1-MCP. Fruits harvested at the MG stage and treated with air for 24 h were used as a control. The change in relative transcript levels of genes in fruits treated with ethylene or 1-MCP are shown, determined by quantitative real time RT-PCR as described above. Asterisks indicate values with a significant difference as determined by Student’s *t*-test (* = p < 0.05, ** = p < 0.01)

To examine the ethylene responsiveness of these *NF-Y* genes, their expression levels were monitored in tomato fruits treated with ethylene or the ethylene receptor inhibitor 1-methylcyclopropene (1-MCP). Specifically, the expression of thirteen candidate ripening related *NF-Y* genes was evaluated in fruit that were harvested at the MG stage and then treated with ethylene or 1-MCP for 24 h, using quantitative RT-PCR analysis. Expression of an ethylene-inducible gene, *ACS2* increased in the ethylene treated fruits, and was suppressed in the 1-MCP treated fruits compared with the control (Fig. 4), thereby confirming the induction or inhibition of ethylene signaling by the treatment. The treatment also significantly increased the expression of eleven *NF-Y* genes (Fig. 4), while the expression of *Solyc01g079870* showed no significant change at the MG stage when treated with ethylene or 1-MCP (Fig. 4c), and the expression of *Solyc07g065500* declined after treatment with 1-MCP but was not significantly affected by the exogenous ethylene (Fig. 4b), which was in accordance with the flat trend during ripening (Fig. 3b). *Solyc03g110860* was the only gene of those tested whose expression was induced by ethylene and decreased to 1-MCP. This pattern was unexpected as we observed that the expression of *Solyc03g110860* decreases during tomato ripening (Fig. 3c).

### TRV-mediated VIGS of *NF-Y* genes affects tomato fruit ripening

We next investigated the functions of the thirteen ripening regulator candidates using TRV-mediated virus induced gene silencing (VIGS), an effective tool to down-regulate gene expression [12] that has proven useful for gene functional characterization in tomato fruits [18, 53, 54]. Except for two pairs of genes (*Solyc03g110860/Solyc06g072040* and *Solyc08g062210/Solyc11g065700*), which were highly homologous and therefore shared the same gene fragment in the VIGS vector, gene specific fragments of each gene, ranging from 400 bp to 500 bp, were selected for VIGS plasmid construction. As a result, a total of 11 specific recombinant plasmids and one positive *pTRV2* vector carrying a truncated *PHYTOENE DESATURASE (tPDS)* gene fragment were used for the VIGS experiments.

**Fig. 5:**
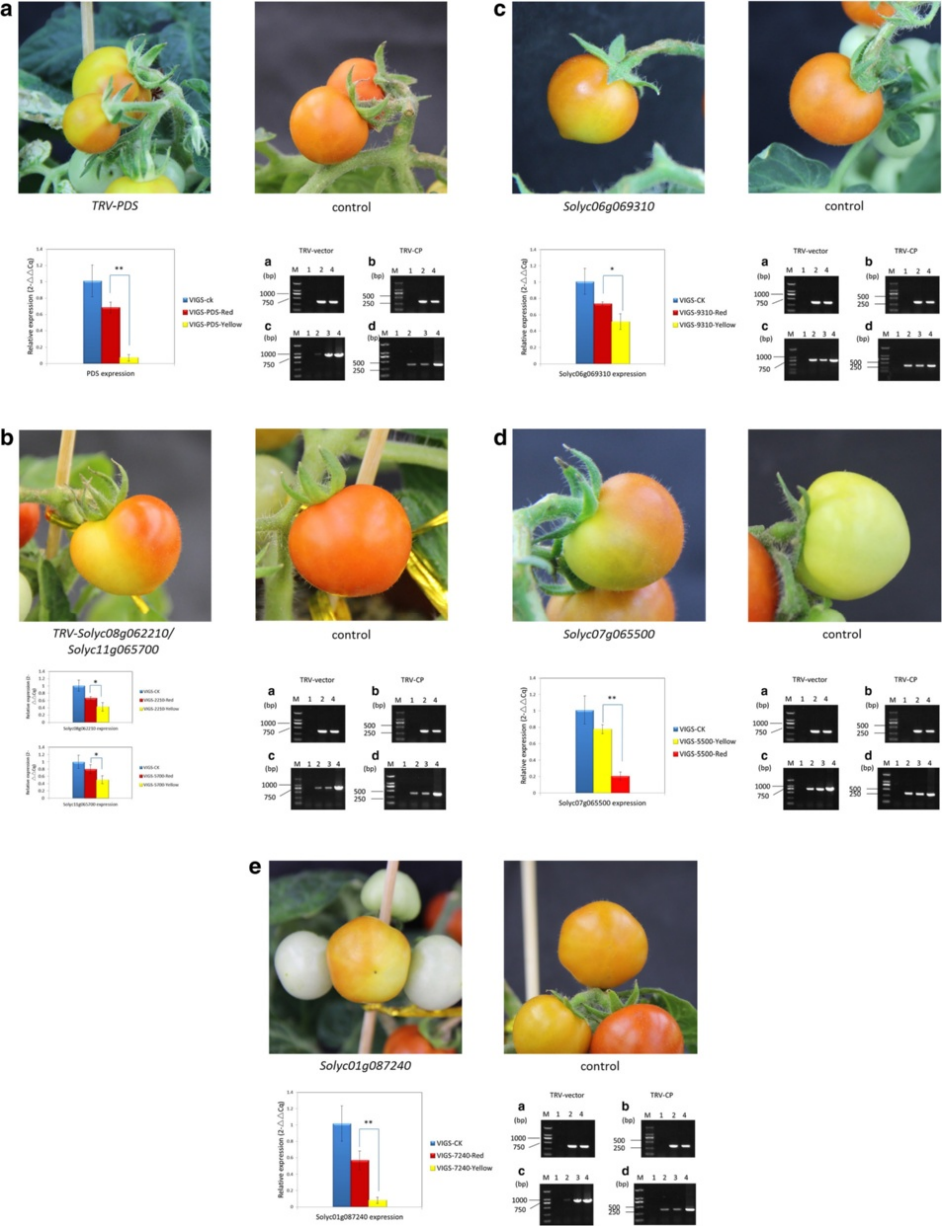
TRV-mediated virus induced gene silencing (VIGS) of genes in tomato fruit. The inflorescence peduncles attached to the fruit were injected with *Agrobacterium tumefaciens* transformed with TRV alone, or with pTRV2 carrying a fragment of a target gene. **a** pTRV-PDS, (**b**) pTRV2-Solyc08g062210/Solyc11g065700, (**c**) pTRV2-Solyc06g069310, (**d**) pTRV2-Solyc07g065500, (**e**) pTRV2-Solyc01g087240. Silencing of the target gene led to a decrease in gene expression, and different fruit color phenotypes compared with plants that were transformed with the empty vector control, which displayed normal ripening fruits (a-e). RNA was extracted from the control and the red and yellow areas of the gene-silenced tomato fruits. After reverse transcription, both the control and silenced samples were assayed for the presence of the TRV virus using PCR with primers specific to the TRV2 vector (**a**, **c**), and a virus coat protein (CP) gene (**b**, **d**). Lane 1 = negative control, Lane 2 = red part of the silenced tomato fruit, lane 3 = yellow part of the silenced tomato fruit, and lane 4 = the positive control. RT-PCR analysis of the target genes in the silenced tomato fruits showed significantly different expression in the yellow areas of the fruits. Asterisks indicate a significant difference as determined by Student’s *t*-test (* = p < 0.05, ** = p < 0.01)

Approximately ten days after infiltration of the fruit stalk with mixed *Agrobacterium strain* GV3101 containing pTRV1 and pTRV2 or one of the twelve recombinant vectors (similar dual viral vectors construction has been reported by [42]), fruits of the infiltrated plants developed an uneven coloring, which was visible for several days. As expected [31], the *PDS* gene-silenced fruit (positive control) displayed a photo-bleached phenotype, which led to a partial yellow coloring of the fruits at the red colored stages (Fig. 5a). Silencing of 5 *NF-Y* genes (*Solyc01g087240, Solyc06g069310, Solyc07g065500* and *Solyc08g062210/Solyc11g065700)* resulted in patchy coloring of the fruit with areas exhibiting different shades of yellow, orange or pink (Fig. 5b to e). In order to ensure the efficiency of the VIGS, the presence of the virus and gene transcript levels were determined (Fig. 5). We observed that mRNA levels of *Solyc01g087240, Solyc06g069310* and *Solyc08g062210/Solyc11g065700* in the yellow colored areas were reduced by approximately 85 %, 30 %, 35 % and 37 %, respectively, compared with the orange tissues, which suggested that the expression of these genes was upregulated during ripening. In contrast, the mRNA levels of *Solyc07g065500* in the red regions were much lower than in the yellow regions (a 74 % reduction), indicating a role in suppressing ripening. Finally, silencing of *Solyc01g087240, Solyc06g069310* and *Solyc07g065500*, resulted in delayed ripening at earlier stages, with a subsequently restored red phenotype at later stages. Only the fruits of plants infiltrated with *pTRV2-t2210/t5700*, which silenced *Solyc08g062210/Solyc11g065700,* displayed patchy coloring until the Red Ripe stage (Additional file 5: Figure S5), which may indicate that simultaneous silencing of these two genes strengthens the effect of the VIGS.

### Subcellular localization of *NF-Y* genes

**Fig. 6:**
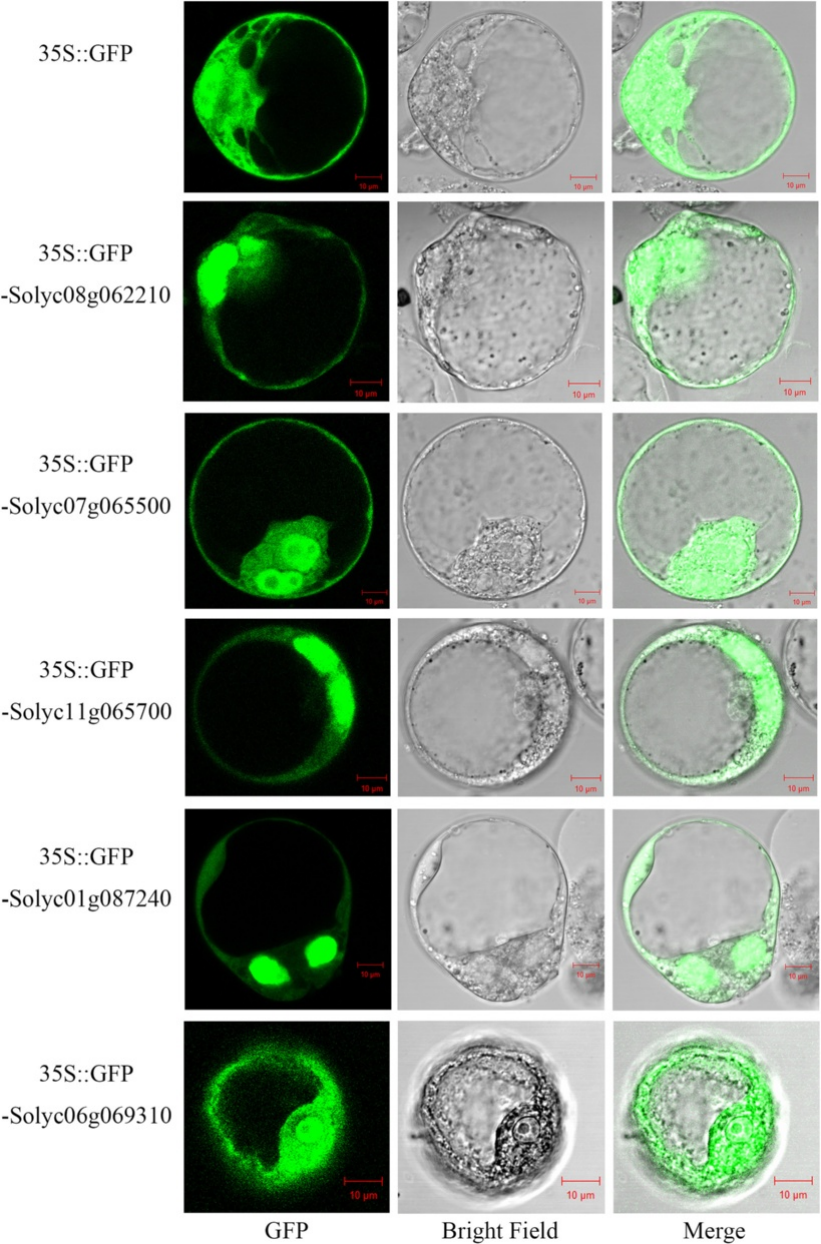
Subcellular localization of *NF-Y* proteins. Tobacco BY2 protoplasts were transiently transformed with 35S-GFP-NF-Y or a GFP empty vector, and fluorescence was observed with a fluorescence microscope

From the VIGS experiments it was concluded that five NF-Y TF genes affected tomato fruit ripening. It is known that TFs regulate the transcription of target genes through binding to specific *cis*-elements in their promoters and that this binding takes place in the nucleus. To assess the subcellular localization of the five TFs, their full-length open reading frames (ORFs) without the stop codon were cloned into a vector in frame with the green fluorescence protein (GFP) reporter gene under the control of the CaMV35S promoter. The resulting constructs and an empty (control) vector were transiently expressed in tobacco BY2 protoplasts. Fluorescence microscopy revealed that TFs with a NF-YA subunit, including Solyc01g087240, Solyc08g062210 and Solyc11g065700 were localized in the nucleus (Fig. 6), which is consistent with the subcellular localization of NF-YA proteins in mammals [26] and *A. thaliana* [22, 71]. One exception to this localization pattern was the wheat TaNF-YA10-1, which was found to be localized in both the nucleus and the cytoplasm [44]. TFs with a NF-YB subunit, including Solyc07g065500 and Solyc06g069310, were localized in the nucleus and cytoplasm (Fig. 6). It is known that NF-YB lacks a nuclear localization signal, and that only when it interacts with NF-YC, can the heterodimers be transported from the cytoplasm to the nucleus [26]. We infer from this result that heterodimers of tomato NF-YB proteins and endogenous tobacco NF-YC TFs likely formed during the transient expression analysis. Taken together, the subcellular localization analysis of the NF-Y TFs supports their proposed role as regulators of target genes.

## Discussion

Based on the current tomato genome sequence and annotations from related databases, including SGN and PlantTFDB, a total of fifty-nine tomato *NF-Y* genes were identified. In contrast with the well-studied functions of *NF-Y* genes in regulating plant growth and development, as well as abiotic and biotic stress responses [4, 36, 57], little is known about their role in fruit ripening. To address this deficiency, we used a combination of bioinformatic analysis and high-throughput VIGS based gene functional screening to assess the potential involvement of fifty-nine predicted tomato *NF-Y* genes in ripening. The bioinformatic analysis included a study of the phylogenetic relationships between *A. thaliana* and tomato NF-Y proteins and an analysis of the chromosomal distribution, encoded protein motifs and exon/intron structure patterns of the tomato *NF-Y* genes. This revealed similarities and conservation of *NF-Y* gene function between *A. thaliana* and tomato. Subsequent analysis of gene expression patterns, coupled with VIGS-based silencing and subcellular localization studies of candidate genes, supported the involvement of a subset of NF-Y TFs in regulating tomato fruit ripening.

Bioinformatic analysis can be used to help predict the biological functions of gene prediction, and one approach is to use phylogenetic relationships among homologs from different species to infer function. As an example, there have been few functional studies of *NF-Y* genes in fleshy fruit development but these genes have been studied in more detail in *A. thaliana* [57], and so we performed a phylogenetic analysis of all the *A. thaliana* and tomato *NF-Y* genes. We reasoned that members of the same clade might have similar specific expression patterns, such as that shown by *AtNF-YB6 (L1L),* which is a critical regulator of embryo development, and is specifically expressed in developing embryos [35]. The tomato homologs, *L1L1* to *L1L13*, were reported to be expressed either in seeds, or in developing fruit, or both [24]. In some cases, similarities in terms of gene structures have also been attributed with biological significance [68]. We observed that genes in group *NF-YB* with only one exon (e.g. *Solyc07g065500*, *Solyc12g006120*, *Solyc01g099320*, *Solyc06g009010* and *Solyc09g074760*) were in the same clade, while genes in another clade (*Solyc03g114400* and *Solyc06g069310*) had six exons. In addition, similar structures were noted for three *NF-YC* gene pairs: *Solyc03g111460*/*Solyc03g111470*, *Solyc00g107050*/ *Solyc11g016910*, and *Solyc11g072150*/ *Solyc01g096710. NF-YA* genes had fewer difference among members in gene structures (Additional file 1: Figure S1).

Using this analysis as a foundation, we next examined the expression patterns of the tomato *NF-Y* genes during ripening to identify those that were specifically expressed, or showed predominant expression, in fruit. Publicly available RNA-seq data, was used to assess the expression of the tomato *NF-Y* genes, which suggested thirteen candidate fruit ripening related regulators. Real-time quantitative RT-PCR analysis, of these thirteen *NF-Y* genes revealed a range of expression pattern during fruit ripening and response to treatments with ethylene or 1-MCP. The expression of some of these genes such as *Solyc11g065700* (Fig. 3a), *Solyc09g007290* (Fig. 3b), *Solyc01g079870* and *Solyc01g096710* (Fig. 3c), was up-regulated during ripening and induced by ethylene (Fig. 4), whose synthesis in system 2 is key to climacteric fruit including tomato [5]. Expression of *Solyc06g069310* was induced by the ethylene treatment (Fig. 4b), which was consistent with its up-regulated expression at the BK stage (Fig. 3b). Other genes, including *Solyc11g068480* (Fig. 3b), *Solyc06g016750* and *Solyc06g072040* (Fig. 3c) were induced by ethylene, but showed relatively minor changes in expressions during ripening, especially at the beginning of the BK stage. We deduced that these might not act as key regulators of ripening, especially when compared to *Solyc08g062210* and *Solyc07g065500*, whose silencing significantly influenced normal ripening (Fig. 5). Expression of some genes, such as *Solyc01g087240* and *Solyc01g006930* (Fig. 3a) was lower at the BK stage compared to MG but was induced by ethylene treatment (Fig. 4), which suggests that ethylene might not be the only factor regulating their expression at onset of ripening. It was reported that *NF-YA* genes are the target of the microRNA169 [52, 58], but it is not known whether miR169 regulates their expression at onset of tomato fruit ripening. Interestingly, expression of *Solyc03g110860* was not only induced by exogenous ethylene but also reduced by 1-MCP (Fig. 4c); however, its down-regulated expression during ripening indicated a negative association with fruit ripening (Fig. 3c). In climacteric fruit, once ripening is initiated, the burst of system 2 ethylene is accompanied with the activation of many ripening related genes, such as *ACS2* and *ACS4*. In this study, expression of *Solyc03g110860* was induced by exogenous but not endogenous ethylene, which may suggest that exogenous ethylene treatments induce stress in tomato fruits. This may be related to the observations that some *NF-YC* genes play a role in responses to abiotic and biotic stress [23, 80]. It is also not known whether some NF-YC proteins whose functions overlap with that those of Solyc03g110860 competitively recruit NF-YA after forming a complex with NF-YB. Plant *NF-Y* genes are known to exhibit functional redundancy (Kuminoto et al., 2010) and this might result in varying responses to ethylene when the fruits start to ripen.

The gene expression patterns suggested candidates for further analysis using VIGS, a powerful tool for functional gene studies of tomato fruit development and ripening [18, 53, 60], which has been used to assess the functions of genes encoding ubiquitin-conjugating enzymes in fruit ripening, such as *SlUBC32 & SlUBC41* [76]. The VIGS studies, together with confirmed subcellular localization of the candidate proteins in nucleus, indicated that NF-Y TFs likely act to regulate tomato fruit ripening. For example, silencing the expression of either *Solyc01g087240, Solyc06g069310* or *Solyc07g065500* resulted in altered fruit pigmentation in the earlier stages of ripening, although the fruits were uniformly red at later stages. For these fruits, the restoration of normal ripe coloration suggests that homologous genes may have compensated for the loss of function of the targeted *NF-Y* genes. Indeed, sequence similarity among the *NF-Y* genes was apparent and the phylogenetic analysis indicated that Solyc11g065700 is closely related to Solyc01g008490, with which it shares 70 % amino acid identity. Additionally, Solyc07g065500/Solyc12g006120, and Solyc03g114400/Solyc06g069310 share 68 % and 89 % AA identity, respectively (data not shown). As a precedent, complementation of gene functions in the context of gene silencing has been reported for *SlUBC32* and *SlUBC41* in the ubiquitination process during tomato fruit ripening [76]. Silencing of *Solyc07g065500* led to accelerated color development in ripening fruit compared to the control, which suggests a role in suppressing ripening. Indeed, the down-regulation of *Solyc07g065500* expression during ripening (Fig. 3b), supports such role at the BK stage. A significant down-regulation tendency was consistent with its proper negative role at BK stage. A similar phenomenon been reported for SlMADS1, the transcript abundance of *SlMADS1* decreases significantly during ripening, and *SlMADS1*-silenced tomato fruits have a reduced ripening time, which suggested that *SlMADS1* is a negative regulator of fruit ripening [13].

Taken together, the results of this study suggest that NF-Y TFs play roles in the transcriptional regulation of tomato fruit ripening. The specific functions and modes of action of the different family members that were highlighted, and the interactions among the NF-Y TFs with different subunits will be the subject of further studies.

## Conclusion

In this work, fifty-nine tomato *NF-Y* genes were identified, and the chromosomal distribution, gene structures, phylogenetic relationship and expression patterns were characterized. Among the fifty-nine *NF-Y* genes, the expressions of thirteen members showed evidence of being related to fruit ripening. Furthermore, we examined their biological functions in ripening regulation using VIGS and subcellular localization studies. We determined that five *NF-Y* genes, including two members of the NF-YB subgroup (*Solyc06g069310, Solyc07g065500*) and three members of the NF-YA subgroup (*Solyc01g087240, Solyc08g062210, Solyc11g065700*), influence ripening. In addition, subcellular localization analyses confirmed the localization of the encoded proteins, fused to the GFP reporter, in the nucleus. We conclude that 5 NF-Y TFs play roles in tomato fruit ripening, and this will provide a platform for further investigation of their biological functions and the evolution of tomato *NF-Y* gene family.

## Methods

### *NF-Y* gene IDs and sequences

Tomato (*S. lycopersicum*) and *A. thaliana NF-Y* gene sequences were obtained from the PlantTFDB database v3.0 (http://planttfdb.cbi.edu.cn/) [25]. The annotated *S. lycopersicum* gene sequences were downloaded from the Sol Genomics Network (SGN, ftp://ftp.solgenomics.net/genomes/Solanum_lycopersicum/annotation/ITAG2.4_release/) database.

### *NF-Y* gene structure and chromosomal location

The tomato *NF-Y* genes were assigned to chromosomes according to the positions given in the SGN database, as were the exon-intron organizations of individual *NF-Y* genes. Gene structures were visualized using Fancy gene v1.4 [61].

### Alignments and phylogenetic analysis of *NF-Y* genes

Tomato and *A. thaliana* NF-Y AA sequences were obtained from the PlantTFDB database v3.0 (http://planttfdb.cbi.edu.cn/) and aligned using the Vector NTI Advance 11.5.1 software (Invitrogen). Alignments of AA sequences of full length NF-Y proteins were made using Clustal *X*2.1 [37]. An unrooted phylogenetic tree was constructed based on the alignments using MEGA4.0 (http://www.megasoftware.net/mega4/mega.html) [73] and the neighbor-joining (NJ) method. The parameters used in the tree construction were Amino: Poisson correction model plus Uniform rates determined by Pairwise deletion and 1,000 bootstraps. The trees were visualized using the online tool, EvolView [81]. Protein motifs were annotated using InterPro 51.0 [49] and visualized in DOG2.0.1 (http://dog.biocuckoo.org/) [62].

### Transcriptome data analysis

The normalized expression levels of tomato genes, based on RNA-seq data, were obtained from The Genome Consortium (TGC) datasets [65]. To visualize the expression patterns of the *NF-Y* genes in different tomato organs, a heat map was created using R Project (http://www.r-project.org/). To generate a heat map of gene expression, the RPKM (Reads Per Kilobase of exon model per Million mapped reads) values of all genes were transformed to log2 (value + 1) to reveal differences in expression levels between observed organs among observed NF-Y members. A cut-off value of 30 (log_2_^31^ in the heat map) was set to define higher expression, based on a significant distribution difference of published RPKM data [50], RPKM values higher than 30 were considered highly expressed, corresponding a value higher than log_2_^31^ in the heat map. Genes with higher expression in fruit were selected for following experiments.

### Plant material and growth conditions

Tomato (cv. Micro-Tom [MT]) seedlings were grown in a greenhouse under long day conditions (16-h light, 8-h dark) at a temperature of 26 °C. For gene expression analysis, organs were collected, frozen in liquid nitrogen and stored at −80 °C until RNA extraction. Three independent samplings were performed.

### Ethylene and 1-MCP treatment

Tomato fruit at the Mature Green (MG) stage were placed into an airtight 1-l plastic container with 100 μl/L ethylene and control fruit were placed in the same containers with air. For the 1-MCP treatment MG fruit were placed into the same airtight containers with 10 ppm of 1-MCP that was generated by dissolving 48 mg of 1-MCP-releasing powder in 50 μl of water [19]. The control fruit were placed into the same container with no powder added to the water. Both the treatments were conducted for 24 h in an incubator under 16-h light and 8-h dark at 25 °C conditions. After the treatment, the fruits were sliced, frozen in liquid nitrogen, and subjected to RNA isolation followed by quantitative RT-PCR. The effect of ethylene or 1-MCP was confirmed by quantitative RT-PCR using a pair of primers for *ACS2* [79]. Three biological replicates were analyzed with each replicate sample being derived from four tomato fruits.

### RNA isolation and real-time quantitative RT-PCR

RNA was isolated from the pericarp of tomato fruits at different developmental stages during ripening: Immature (IM), Mature Green (MG), Breaker (BK), Pink (PK) and Red Ripe (RR) stages, which occurred at on average at 37, 42, 46, 51 and 56 days post-anthesis (DPA), respectively. Total RNA extraction was carried out using DeTRNa reagent (EarthOx, CA, USA) according to the manufacturer’s protocol, and RNA integrity was verified by 1.5 % agar gel electrophoresis. Genomic DNA was removed from the RNA preparations by digestion with DNase I (TaKaRa, China), and RNA quality and quantity was confirmed by spectrophotometry (Thermo Scientific, NanoDrop™ 1000). RNA was reverse-transcribed into cDNA using M-MLV Reverse Transcriptase (Promega, USA) according to the manufacturer's instructions. Real-time quantitative RT-PCR was conducted using TransStart Top Green qPCR SuperMix (Transgen, China) with a real-time PCR System CFX96 (Bio-Rad, CA, USA). The reactions were performed with the following cycling profile: 95 °C for 30 s, 40 cycles at 95 °C for 5 s, and 60 °C for 20s. Melting curve analysis was performed to verify the specificity of the amplification for each primer pair. The tomato *ACTIN* gene (*Solyc03g078400*) was used as an internal reference gene, and tomato ripening-related *ACS2* (*Solyc01g095080*) as positive control. Relative gene expression values were calculated using the 2^-△△Ct^ method [43]. Three biological replicates were analyzed with each replicate sample being derived from four tomato fruits. A pairwise Student’s *t* test was performed to determine whether the qRT-PCR results were statistically different between two samples (*P < 0.05;**P < 0.01). Primers designed for Real-time quantitative RT-PCR are listed in Additional file 6: Table S1.

### Plasmid construction for VIGS

The pTRV1 and pTRV2 VIGS vectors have been previously described [42]. In order to improve the cloning efficiency, we used a Cloning and Assembly Kit (Transgen, China). All the primers designed for PCR-amplification had a 15-bp overlap with the linearized pTRV2 vector and contained a *Bam*HI restriction site. Since *Solyc03g110860* and *Solyc06g072040* are highly homologous, they shared the same gene fragment in the VIGS vector, as did *Solyc08g062210* and *Solyc11g065700*. The primers are listed in Additional file 7: Table S2.

### TRV mediated VIGS in tomato fruit

*Agrobacterium tumefaciens* strain GV3101 containing pTRV1 or pTRV2 and its derivatives were used for the VIGS experiments. GV3101 containing the TRV-VIGS vectors were grown at 28 °C in LB medium containing 10 mM MES buffer (pH5.6) and 20 mM acetosyringone with appropriate antibiotics (gentamicin and rifampicin for GV3101, kanamycin for pTRV1 or pTRV2). After overnight culturing (28 °C, 200 rpm), *A. tumefaciens* cells were harvested and resuspended in infiltration buffer (10 mM MgCl2, 10 mM MES [pH 5.6] and 150 mM acetosyringone) to a final OD_600_ of 2.0 (for both pTRV1 or pTRV2 and its derivatives). *A. tumefaciens* containing pTRV1 and pTRV2 or the recombinant vectors were mixed in a 1:1 ratio, and left for 3-5 h at room temperature before infiltration, as previously described [18]. The tomato inflorescence pedicels attached to the fruit were injected with cultures of *A. tumefaciens* harboring the vectors, using a 1-ml syringe. For biological replicates, at least 12 individual Micro-Tom tomato plants were injected for each gene silenced.

### VIGS phenotype analysis, virus assay and quantitative RT-PCR

To detect the accumulation of the virus in the fruit, total RNA was extracted (as above) from different regions of tomato fruit showing different external coloration, and reverse-transcribed into cDNA using the M-MLV Reverse Transcriptase (Promega, USA) with a TRV-RNA2-specific primer, 5’-GGGCGTAATAACGCTTACGTAGGC-3’. The RNA2 cDNA of TRV was amplified with the RNA2-specific primers (GenBank accession number AF406991), 5’-CGGTCTAGAGGCACTCAACTTTATAAACC-3’ and 5’-CGGGGATCCCTTCAGTTTTCTGTCAAACC-3’. The RNA2 cDNA of the viral coat protein (CP) was amplified with the primers, 5’-CTGACTTGATGGACGATTCTT-3’ and 5’-TGTTCGCCTTGGTAGTAGTA-3’. To detect the silencing efficiency of specific genes, the isolated total RNA was reverse-transcribed into cDNA using the M-MLV Reverse Transcriptase (Promega, USA) with an oligo (dT)_18_ primer. Real-time quantitative RT-PCR was performed to analyze gene expression patterns, as above. A pairwise Student’s *t* test was performed to determine whether the qRT-PCR results were statistically different between two samples (* = P < 0.05;** = P < 0.01). The primers used are listed in Additional file 8: Table S3. Each analysis was analyzed separately for three different fruits.

### Subcellular localization

The coding sequence (CDS) of the five *NF-Y* genes without the stop codon was amplified by PCR (primers are listed in Additional file 9: Table S4, CDS are listed in Additional file 10: Table S5) and sub-cloned into the pBI221-GFP vector, in frame with the green fluorescent protein (GFP) sequence, resulting in a set of 35S::GFP-NF-Y vectors. These fusion constructs and the control GFP vector were transformed into tobacco (*Nicotiana tabacum*) BY-2 suspension culture cell protoplasts using the polyethylene glycol (PEG) method [2, 67]. GFP fluorescence was observed with a fluorescence microscope (Zeiss7 10-3 channel). All transient expression assays were repeated at least three times.

### Availability of supporting data

The RNA-seq data, are downloaded from The Genome Consortium (TGC) datasets, and can be obtained from http://www.nature.com/nature/journal/v485/n7400/extref/nature11119-s3.zip.

Other data supporting the results of this article are included within the article and its additional files.

## Additional files

Additional file 1: Figure S1. Chromosome distribution of *NF-Y* genes in the tomato genome. The gene IDs below denotes each specific *NF-Y* gene. The position of each gene on the bar denotes the relative location on the corresponding chromosome. Arrows following each gene indicate the orientation of the gene. (JPG 7258 kb) Additional file 2: Figure S2. Exon-intron structure of NF-Y genes in tomato. The exon and intron distribution of each gene is shown. Rectangles represent the location and length of exons and broken lines indicate the location and length of introns. Arrows at the end of the last exon indicate the direction of the gene on the chromosome. (JPG 7098 kb) Additional file 3: Figure S3. Alignment of tomato and *A. thaliana* NF-YA domains. Numbers in parentheses correspond to the start and end sites of the NF-YA domain in the given protein. The amino acids that are predicted to be important for DNA binding and subunit interaction are based on Quach et al. [59]. The three boxes in red are the basic residual clusters required for nuclear targeting ([56]. (JPG 8734 kb) Additional file 4: Figure S4. Motif composition of tomato NF-Y proteins. A schematic representation of motif composition of tomato NF-Y proteins is shown based on InterPro protein sequence analysis and classification, including NF-YA (a), NF-YB (b) and NF-YC (c) subunits. (JPG 9995 kb) Additional file 5: Figure S5. TRV-mediated VIGS of *Solyc08g062210/Solyc11g065700* in tomato fruit. External fruit color changes induced by VIGS infiltration were maintained until the Red Ripe stage. (JPG 7768 kb) Additional file 6: Table S1. Primers for real time qPCR analysis of expression during fruit ripening. (PDF 97 kb) Additional file 7: Table S2. Primers for VIGS plasmid construction. (PDF 142 kb) Additional file 8: Table S3. Primers for real time qPCR analysis of the VIGS assay. (PDF 84 kb) Additional file 9: Table S4. Primers for plasmid construction used in subcellular localization assays. (PDF 79 kb) Additional file 10: Table S5. CDS of *NF-Y* genes in subcellular localization. The CDS of each *NF-Y* gene was cloned as a fusion with EGFP with a deletion of termination codon. (PDF 118 kb)

## Abbreviations

1-MCP: 1-methylcyclopropene
AA: amino acid
ARF: auxin respons factor
BK: Breaker
CDS: coding sequence
ERF: ethylene response factor
GFP: green fluorescence protein
IM: immature
L1L: LEC1-LIKE
MG: mature green
NF-Y: nuclear factor Y
NJ: neighbor-joining
ORFs: open reading frames
PEG: polyethylene glycol
PK: pink
RPKM: reads per kilobase of exon model per million mapped reads
RR: red ripe
SGN: Sol Genomics Network
TFs: transcription factors
TGC: the genome consortium
tPDS: truncated PHYTOENE DESATURASE
VIGS: virus induced gene silencing
