# Supplementary material for: Genome-wide analysis of tomato NF-Y factors and their role in fruit ripening
Source: BMC Genomics. 2016 Jan 7;17:36. doi: 10.1186/s12864-015-2334-2 (PMC4705811; doi:10.1186/s12864-015-2334-2)
Supplement: Additional file 6: Table S1. — Primers for real time qPCR analysis of expression during fruit ripening. (PDF 97 kb) [file 12864_2015_2334_MOESM6_ESM.pdf]

Primers for real time PCR analysis in ripening process of tomato fruit

	primers	sequences (5'to3')	bases	length
1	Solyc01g006930-FS	TTTCGTGTCTGGTAAGTGGGTTTGC	25	123
	Solyc01g006930-RA	GGTTGTAGCACGGATGAGGTGAGGC	25	
2	Solyc01g079870-FS	AAAGCGGATGAAGATGTTAGGATGA	25	69
	Solyc01g079870-RA	TCTCACAGGCACGAGCAAATACGAC	25	
3	Solyc01g087240-FS	GGTCAGCCCAGCATGGTTCCGTCTC	25	99
	Solyc01g087240-RA	AAGAATGAATTGCCTGAGCATAGCC	25	
4	Solyc01g096710-FS	GGCAGATGAAATAACAAACAAATGG	25	131
	Solyc01g096710-RA	GACAACTAAGAAATAGACGGACACT	25	
5	Solyc03g110860-FS	TACCAACGCAAGGAAATCGAACAGG	25	116
	Solyc03g110860-RA	GCTTCAGCGGAGATCATACGGACAT	25	
6	Solyc06g016750-FS	GATTTCGTCATACCATCTTCCAGTG	25	132
	Solyc06g016750-RA	CTCCTCCTCTTCTTCGTCTTCTTTG	25	
7	Solyc06g069310-FS	AAGTGCAGTAATGGAGCTGAAATG	24	131
	Solyc06g069310-RA	TCTGCTTCTGAATCTTGCTGCTTGG	25	
8	Solyc06g072040-FS	CAGACTCCATACCACCATCTCCTTC	25	91
	Solyc06g072040-RA	CATTGACTTGCTCGATTTCTTGACG	25	
9	Solyc07g065500-FS	GGGAAGCATCAGATAAGTGTCAAAG	25	70
	Solyc07g065500-RA	ATTGCCCACAACAGATCATCACCAT	25	
10	Solyc08g062210-FS	CAAGCCTTCATCTCCAATGCACGAC	25	65
	Solyc08g062210-RA	TAGATAAGTTGCCACCTGCCTGTCG	25	
11	Solyc09g007290-FS	TTGCGAAGGATGCTAAGGAGACTGT	25	72
	Solyc09g007290-RA	TTGCCTCGCTAGTAATGAAGCTGAT	25	
12	Solyc11g065700-FS	CTGGTTCTGTGAGTTCATCGGGTTC	25	129
	Solyc11g065700-RA	GATTGCCATAACTGCTACCATTTG	24	
13	Solyc11g068480-FS	ACTTCTTCGTGACTCGCTTCTTGCT	25	111
	Solyc11g068480-RA	TTTATCGTTTGCCTCTTAGACTCCT	25	
14	actin-FS	CAGCAGATGTGGATCTCAAA	20	59
	actin-RA	CTGTGGACAATGGAAGGAC	19	
