# Supplementary material for: Genome-wide analysis of tomato NF-Y factors and their role in fruit ripening
Source: BMC Genomics. 2016 Jan 7;17:36. doi: 10.1186/s12864-015-2334-2 (PMC4705811; doi:10.1186/s12864-015-2334-2)
Supplement: Additional file 7: Table S2. — Primers for VIGS plasmid construction. (PDF 142 kb) [file 12864_2015_2334_MOESM7_ESM.pdf]

Primers of plasmid construction for VIGS

	vector	primers	sequences (5'to3')	bases	location	length
1	*pTRV2* -tPDS	Forward	GCTCTAGA GACAGGGTGACAGATGAGGT	28	1084–1559	476
		Backward	CGAGCTCTCTTTCCAGTCTTCAGGCA	26		
2	*pTRV2* -t6930	Forward	GCCTCCATGGGGATCC GAAGGCTATATTTCATTGGTC	37	1–500	500
		Backward	GCTCGGTACCGGATCC GGAAGTTTTTGCCCATTTGC	36		
3	*pTRV2* -t9870	Forward	GCCTCCATGGGGATCC GGGCTTCCCTCGATGTGA	34	33–490	458
		Backward	GCTCGGTACCGGATCC ATTCTTGAAATCAGTAACATG[illegible]	38		
4	*pTRV2* -t7240	Forward	GCCTCCATGGGGATCC CATATGTATGTAATAGACACA[illegible]	38	24–500	477
		Backward	GCTCGGTACCGGATCC AGTCATCATTCCCCCATAC	35		
					2–500	499
5	*pTRV2* -t6710	Forward	GCCTCCATGGGGATCC AATACCTTCGTTTTGCACTTT	37		
		Backward	GCTCGGTACCGGATCC TAGCAACTTTCCTTCTTTTTG	37		
6	*pTRV2* -t0860/t2040	Forward	GCCTCCATGGGGATCC CATTACGCGGACTGATATTT	36	444–903	460
		Backward	GCTCGGTACCGGATCC ATTCAAGTCATTACAGTTAAG[illegible]	38		
7	*pTRV2* -t6750	Forward	GCCTCCATGGGGATCC GAAGGAAGGAGATGAAGAG	35	952–1411	460
		Backward	GCTCGGTACCGGATCC TCTGGGTCGTTTGGAAACA	35		
8	*pTRV2* -t9310	Forward	GCCTCCATGGGGATCC GAAACGAACAATCGCACCC	35	284–687	404
		Backward	GCTCGGTACCGGATCC GAACTAGAGCCCTCACAAG	35		
9	*pTRV2* -t5500	Forward	GCCTCCATGGGGATCC GATAATGAATCAGGAGGACA	36	13–495	483
		Backward	GCTCGGTACCGGATCC AGCATTCTCCCCCGTATG	34		
10	*pTRV2* -t2210/t5700	Forward	GCCTCCATGGGGATCC GTGTAAAGCTTCTAGAGCAC	36	4–492	489
		Backward	GCTCGGTACCGGATCC ACGAGCCTTCCAAAGTAAG	35		
11	*pTRV2* -t7290	Forward	GCCTCCATGGGGATCC CAAGTGCCAGAGAGAGAAA	35	237–649	413
		Backward	GCTCGGTACCGGATCC GACATGGCACTCACTAGAG	35		
12	*pTRV2* -t8480	Forward	GCCTCCATGGGGATCC ATGGCGGAGATAGAGAAGC	35	1–475	475
		Backward	GCTCGGTACCGGATCC CTTCATTTTCGTTATCCGAG	36		
