# Supplementary material for: Genome-wide analysis of tomato NF-Y factors and their role in fruit ripening
Source: BMC Genomics. 2016 Jan 7;17:36. doi: 10.1186/s12864-015-2334-2 (PMC4705811; doi:10.1186/s12864-015-2334-2)
Supplement: Additional file 8: Table S3. — Primers for real time qPCR analysis of the VIGS assay. (PDF 84 kb) [file 12864_2015_2334_MOESM8_ESM.pdf]

Primers for real time PCR analysis in VIGS assay

	primers	sequences (5'to3')	bases	length
1	Solyc08g062210-FS	CAAGCCTTCATCTCCAATGCACGAC	25	65
	Solyc08g062210-RA	TAGATAAGTTGCCACCTGCCTGTCG	25	
2	Solyc07g065500-FS	TCAACATCATCAAGGACACGGGTAT	25	112
	Solyc07g065500-RA	TTGCCTCCCAACGTCAGGAAACCGC	25	
3	Solyc11g065700-FS	CTGGTTCTGTGAGTTCATCGGGTTC	25	129
	Solyc11g065700-RA	GATTGCCATAACTGCTACCATTTG	24	
4	Solyc01g087240-FS	GGTCAGCCCAGCATGGTTCCGTCTC	25	99
	Solyc01g087240-RA	AAGAATGAATTGCCTGAGCATAGCC	25	
5	Solyc06g069310-FS	TTGTCCCCTAAAGTTTCTTGTCAGT	25	74
	Solyc06g069310-RA	GTGAGCTAATGGCCAATTCAGTGAC	25	
6	PDS-FS	AGTTAGTCGGAGTACCTGTG	20	94
	PDS-RA	AGTGAGCTTCTGCTGAAGAG	20	
7	actin-FS	CAGCAGATGTGGATCTCAAA	20	59
	actin-RA	CTGTGGACAATGGAAGGAC	19	
