# Supplementary material for: Genome-wide analysis of tomato NF-Y factors and their role in fruit ripening
Source: BMC Genomics. 2016 Jan 7;17:36. doi: 10.1186/s12864-015-2334-2 (PMC4705811; doi:10.1186/s12864-015-2334-2)
Supplement: Additional file 9: Table S4. — Primers for plasmid construction used in subcellular localization assays. (PDF 79 kb) [file 12864_2015_2334_MOESM9_ESM.pdf]

Primers of plasmid construction for subcellular localization

	primers	sequences (5'to3')	bases	length
1	Solyc08g062210-FS	CGGGATCC ATGCTAAGTTTCTCAAAGAAAGGTG	33	975
	Solyc08g062210-RA	ACGCGTCGAC GGTTCCAACATGCAGGAAGTCTTCA	35	
2	Solyc07g065500-FS	CGGGATCC ATGGCGGATTCGGATAATGAATCAG	33	546
	Solyc07g065500-RA	ACGCGTCGAC CCTTTGCCTCCCAACGTCAGGAAAC	35	
3	Solyc11g065700-FS	CGGGATCC ATGCCATCAAATTCCAAAAGCACAA	33	900
	Solyc11g065700-RA	ACGCGTCGAC CTTGACAGAAAAACCTCCTTCCCTG	35	
4	Solyc01g087240-FS	ACGCGTCGAC ATGCCTACTATTGCTAAACATGATG	35	909
	Solyc01g087240-RA	CCCAAGCTT ATTGCTGGAAGCTGCCCCCTGAGAA	34	
5	Solyc06g069310-FS	CGGGATCC ATGTTGCCCCCAGATGTTCGTGTTG	33	393
	Solyc06g069310-RA	ACGCGTCGAC GCTATTCAAGGTTTGCTCTGCTTCT	35	
