# Supplementary material for: Genome-wide analysis of tomato NF-Y factors and their role in fruit ripening
Source: BMC Genomics. 2016 Jan 7;17:36. doi: 10.1186/s12864-015-2334-2 (PMC4705811; doi:10.1186/s12864-015-2334-2)
Supplement: Additional file 10: Table S5. — CDS of NF-Y genes in subcellular localization. The CDS of each NF-Y gene was cloned as a fusion with EGFP with a deletion of termination codon. (PDF 118 kb) [file 12864_2015_2334_MOESM10_ESM.pdf]

CDS of *NF-Y* genes in subcellular localization

	Gene ID	Length(bp)	CDS
1	Solyc08g062210	975	ATGCTAAGTTTCTCAAAGAAAGGTGGCCCTGGAAAAGAAGGTCAATCTTTTGCTCCTTTGTTTGTGAGTTGCTCATCTATG<br>TGGAATTCCAGCGACAAAGCAGAATATGCTCCCTCTAAGAAGGTGCTGACCGGAGTGGAATCTGCATTGGAACATCACTCC<br>GACATCAAGAAATCAGAATGCCAGTTCCAAGATCAGGATTCAACGTCTACTCTGTCAACTGGTCAATCTAATCATGTGGAG<br>GCCGCAATGGGAAAAAGCAATACTGTTCTTCAAAATGTTGCAGTTCATCCAGGTTGGGGTGGAACTTATGAGGTGCAAGCA<br>GAGAGTGGAACAAACGCATCCCGATCAGGCGAAAGTGACACCAACACTCTCCCCCAGCCACAAGTGAATCATAATCATCCA<br>ATGGCTTGCGTATCCTACCCTTTCGCTGACACTTACTTTGGAAGGCTCGTGACTGCTTATGGATCAAATGCCATCATTTAT<br>CCTCAAATGGTTGGTGTCACCTCTACTAGAGTTGCACTTCCTCTTGAATGCACCGAGAGCTTGCCCATTTATGTGAATGCG<br>AAACAATACAGTGCTATTCTCAAAAGGCGACAGGTCCGTGCCAAGCTAGAGGCTCAAAATAAGCTTGTCAAAGACAGAAAG<br>CCATATCTTCACGAGTCTCGACATCGTCATGCAATGAAGAGAGCTAGGGGTTCTGGAGGACGCTTTTTGAACACAAAGAAT<br>ATGCAGGAATCCAAGCCTTCATCTCCAATGCACGACAGAAATATCTTTAAGCGACAGGCAGGTGGCAACTTATCTAGTTCC<br>ATGGTTCAGCACTCAGAGAGTGGTAGTTGGGGGACTTCCACCCAATCTGGTTCTGATGTGACAAGTATCTTCAGTGGTGAC<br>AACATGTTCCAGCAACCAGAGTTCAGAGTCTCTGGCTTCCCTTTTCACATTCAGGAAGCTGAAGACTTCCTGCATGTTGGA<br>ACC
2	Solyc07g065500	546	ATGGCGGATTCGGATAATGAATCAGGAGGACATAGAGATAACAGTAACATTGAGAGTTCCCTAAGAGAACAAGACAGGTTC<br>CTTCCCATAGCAAATGTAAGCAGAATCATGAAGAAAGCTTTACCAGCTAACGCGAAAATCTCAAAAGATGCTAAGGAGGTA<br>GTTCAAGAATGTGTTTCTGAATTCATAAGTTTCATCACAGGGGAAGCATCAGATAAGTGTCAAAGAGAAAAGAGAAAGACA<br>ATCAATGGTGATGATCTGTTGTGGGCAATGACAACTCTTGGTTTTGAAGAATACATTGAGCCACTCAAGATTTATTTGCAG<br>AGGTTTAGGGATTTGGAAGGGCAAAAAAGTGGTGTCTCTGGAGAGAAGGATCATAGTGGATCAGTGGGTTATGTTGAGGAC<br>TACCATGGCATGATGATGATGGGGAGTCAACATCATCAAGGACACGGGTATGGCACCGGTGTATACAATCATCATACGGGG<br>GAGAATGCTGCAGGGGTTGGTACAGGAGGGTCGCGGTTTCCTGACGTTGGGAGGCAAAGG
3	Solyc11g065700	900	ATGCCATCAAATTCCAAAAGCACAAATCGGGAGGAAACCAATGCATATAATGTTCCACGTTCCACTGTATATCCTGAACCT<br>TGGTGGAATGGTGCTGTTTATACTCCTGTTTCCCCTGGGTTGATGCGGGAAAATGCATCAGATTCATCATCATTGGAACAA<br>TCCGTGGATGGCCAATCTCAGTCTGATGGTGGAATCAATGAGGAAGATGATGATGCTCCTGAAAAATCACAAAGTGTCGTA<br>CCTTTGCATGCAGATGGGAGTTATGGGAAGGCGGATCAGAATTTTCAGCCAGCTGCTCCAGCCATACCTCCAAGACTTGAT<br>GGAAGCCTAGCACAGCCCCAGCAGCTTGAACTTGTTGGACACTCTATTGCGTGTGCTCCAAACCCATATGTTGATCCATAC<br>TATGGCGGGATGATGACAGCTTTTGGCCAGCCTTTGGTTCCTCCTCATGTACTTGATATGCACTATGCAAGGATGCCCTTG<br>CCACAAGAAATGGCTCAAGAACCAGTTTATGTTAATGCTAAGCAGTATCGAAGGATCCTGCAGCGAAGACAGTCACGTGCT<br>AAAGCAGAACTTGAAAAGAAGCAAATAAAGGGTAGAAAGCCATATCTTCACGAGTCTCGACATCAGCATGCACTGAGGAGG<br>GTAAGGGCCTCGGGTGGACGTTTTGCCAAAAAGACAGATGCTTCTAAGGGTACTGGTTCTGTGAGTTCATCGGGTTCTGAA<br>CCTTTGCAGTTCAATGCTGCTGATATTCAAAAGAGGAATGAAAATGGAAGGTTGGCCGAGCTTCAGCAGTCTTATTCAAAT<br>GGTAGCAGTTATGGCAATCAAAGTAGCTTTCAAGAATCAAAGGATGAGTACCAGTCTGCTGAAAGCAGGGAAGGAGGTTTT<br>TCTGTCAAG
4	Solyc01g087240	909	ATGCCTACTATTGCTAAACATGATGGTCGGCAGCTGGAGACTGGGCTGCGAATTACCTCACTATCAACTACCCAAATGCAA<br>CCATGGTGGCACGGTTATGGTGATAATACTATGCCTTTGGCTAGTGAGAATGCCGTTGCCCAGGAGAAATCTGAAGGTGGC<br>AACCAGGATAAAGAAACAAAGGCTCTAGCCATGGAATCTGGATCAGATGGAAATAACGAGCAATACAAGCAGCACCTCAAG<br>CATTTTGCACCCACAACTGCTGCCATCATGGCTGAGCAGCAGAAGGAACTCACAGGTCATTCAGCTATGTTGGCGTCATAT<br>CCATATCCAGATATGCAGTATGGGGGAATGATGACTTATGGCGCACCTGTACATCCCCACTTATTTGAAATTCATCATGCC<br>CGAATGCCTTTGCCTCTCGACATGGAAGAGGAACCTGTCTACGTGAATGCAAAACAGTACCATGGAATATTAAGGCGAAGA<br>CAGATAAGAGCTAAAGCAGAGCTTGAGAGGAAAGCTATTAAAGCGAGAAAGCCATATCTGCATGAATCTCGGCATCAGCAT<br>GCTATGAGAAGGGCAAGGGGAACTGGAGGCCGCTTTCTGAATACTAAAAAGCTCAATGACATGGACTGTACACCAACGGAG<br>ACTCAAAAATATGGGGCAACCATTGCAACTCACTCTGGCAACTCTTCTGGTTCCGGGAGTTCTGATCAAGGGGGAAAGGAA<br>GGATCTACAGTTCAAGACATGCACAAAGGACACAGCCAGAGTTTCACAACTGGCAATGGCCATGGTTCATCTGTGTATTTT<br>TCCGCGAGCAGTGGAAGTGAGCAAGGGAATGGCCATTACGGTCATGGAAGTTGGAGCCTGCTGGTCAATCAGGCTTCTCAG<br>GGGGCAGCTTCCAGCAAT
5	Solyc06g069310	393	ATGTTGCCCCCAGATGTTCGTGTTGCCCGAGATACTCAAGATCTTTTGATTGAATGTTGTGTAGAGTTCATTAATCTTATC<br>TCATCAGAATCCAATGAAGTTTGTAATAGAGAAGAGAAACGAACAATCGCACCCGAACATGTACTCAAGGCTTTACAGGTT<br>CTTGGTTTTGGGGAATATATTGAAGAAGTCTATGCTGCATATGAACAACACAAGCTAGAGACCATGGACACTGTGAGAGCT<br>GGAAAGTGCAGTAATGGAGCTGAAATGACTGAAGAAGAAGCATTAGCTGAGCAACAGAGGATGTTTGCTGAGGCGCGTGCA<br>AGGATGAATGGTGGTGTTACAGGTCCCCCCAAGCAGCAAGATTCAGAAGCAGAGCAAACCTTGAATAGC
